# The impact of person-organization value fit on organizational level citizenship behavior

**DOI:** 10.3389/fpsyg.2026.1699506

**Published:** 2026-01-22

**Authors:** Yiyi Chen, Junaidah Yusof, Yue Liang

**Affiliations:** 1 School of Human Resource Development and Psychology, Faculty of Social Sciences and Humanities, Universiti Teknologi Malaysia, Johor Bahru, Malaysia; 2 Azman Hashim International Business School, Universiti Teknologi Malaysia, Kuala Lumpur, Malaysia

**Keywords:** career commitment, organizational citizenship behavior-organization, psychological needs fulfillment, self-determination theory, value fit

## Abstract

Millennial and Gen Z employees are often unwilling to take on additional job responsibilities, which can be attributed to their high levels of self-awareness and diminished collective consciousness. Based on structural equation modeling, we conducted a survey and data analysis involving 639 Chinese Millennial and Gen Z employees born after 1990 from diverse industries and institutions. In line with the key tenets of self-determination theory, the findings indicated that comfort and security, competence and growth, and status and independence dimensions of person-organization value fit positively influence employees’ organizational citizenship behavior toward the organization by fulfilling their basic psychological needs. Moreover, we discovered that this impact is more significant when their commitment to careers is higher. Theoretical and practical implications are discussed, and future research directions are recommended.

## 1 Introduction

Employees are critical stakeholders in an organization, playing an essential role in organizational effectiveness (Im et al., 2016). Lee and Ha-Brookshire (2018) argued that the success of any organization is essentially contingent upon the performance of its employees, insofar as they exert effort exceeding the expectations placed upon them within the workplace. This is especially true in today’s dynamic world of fierce competition, organizations can only survive and thrive by efficiently utilizing their human capital (Huang et al., 2019). Organizational citizenship behavior (OCB) is a typical behavioral outcome of employees, involving out-of-role actions, which can enhance the organization’s capacity to adapt to environmental changes (Al-Madadha et al., 2021), ultimately leading to improved organizational effectiveness and efficiency (Sedlářík et al., 2024). Fostering OCB within companies has been identified by researchers as an effective approach to achieving organizational success and sustainability goals (Servaes et al., 2023; Srivastava et al., 2025).

The Millennial and Gen Z employees have emerged as a crucial demographic in the contemporary workplace and are poised to evolve into the principal driving force of enterprises within the next decade or two. However, one frequently observed workplace trait of the younger generation is their strong self-awareness and weak collective consciousness (e.g., Liu et al., 2023), which gives them a clear boundary of their job responsibility and a lack of willingness to engage in behaviors outside their roles. This will influence the organization’s success, as it relies on the extra-role actions of employees (Lee and Ha-Brookshire, 2018). Therefore, delving into the determinants and underlying mechanisms that impact the organizational citizenship behavior toward the organization (OCBO) of this group is of great research significance for both theoretical and practical implications.

Individual employees are a fundamental component of any organization, and their behaviors are inevitably shaped by a complex web of personal, contextual, and interpersonal factors (Afsar and Badir, 2016). One critical aspect of this phenomenon is the person-organization fit (PO fit), which is a factor in predicting individuals’ positive attitudes and behaviors (Na-Nan et al., 2025). PO fit leads to “behaviors motivated by assisting the organization, not just performing a job” (Kristof-Brown and Guay, 2011, p. 34). According to scholars, value congruence is the most widely recognized definition of PO fit (Chung, 2017), and the most commonly studied form of PO fit (Subramanian et al., 2023), which denotes the extent of similarity between an individual’s personal values and the values embraced by the organization.

Existing literature has substantiated that person-organization value fit (POVF) exerts a favorable influence on the attitudes and behaviors of employees. Attitudinal outcomes include organizational commitment (Oyelakin et al., 2021), job satisfaction (Soares et al., 2024), work engagement (Vila-Vázquez et al., 2023), behavioral consequences include job performance (Rajper et al., 2020), turnover (Hoffman and Woehr, 2006), and OCB (Zoghbi-Manrique-de-Lara et al., 2023). When an individual’s values align with the values of the organization they work for, it creates a positive impression of the organization and results in higher evaluations. Employees are more likely to share emotional connections and trust with the organization, leading to beneficial behaviors for the organization (Zhan et al., 2017). Gould-Williams et al. (2015) indicated that PO fit has significant positive associations with OCBs. Ashfaq and Hamid (2021) examined the positive influence of value fit on OCBO through the impact mechanism of work engagement. Zoghbi-Manrique-de-Lara et al. (2023) revealed positive effects of value fit on OCBO on the contributory role of employees’ compassion at work as a mediator.

Despite existing literature having demonstrated empirical evidence indicating that the congruence of values between employees and organizations positively impacts OCBO, some issues still require further research. Firstly, most existing studies have solely focused on investigating the impact of POVF as a unidimensional construct. Thus, for the younger generations of employees, there is a lack of research examining the differential impacts of value congruence across various dimensions on their behaviors. Secondly, previous research has yet to thoroughly investigate the mechanisms by which PO fit influences OCB (Ashfaq and Hamid, 2021), particularly from the motivational and psychological perspectives. As a typical motivation theory, self-determination theory (SDT) (Ryan and Deci, 2000) suggests that the satisfaction of basic psychological needs is a pivotal link between the environment, such as the degree of value congruence, and individual motivation and behavior (Zhang et al., 2010). However, existing research examining the association between POVF and OCBO has primarily overlooked the mediator of psychological needs fulfillment (PNF).

Moreover, there is a conspicuous dearth of exploration of the boundary conditions of the correlation between POVF and OCBO in existing literature. Zhu et al. (2021) argued that employees tend to develop more commitment to their own careers than to their organizations, particularly among Millennial employees (De Hauw and De Vos, 2010). Employees who exhibit high levels of career commitment are more likely to be motivated to achieve job-related goals, perform at higher levels, and contribute to organizational success (Ahmed, 2019). However, as far as current knowledge extends, no empirical studies regarding the relationship between POVF and OCBO have been conducted to investigate the potential moderating influence of career commitment (CC). As such, this area of inquiry remains underexplored.

**FIGURE 1:**
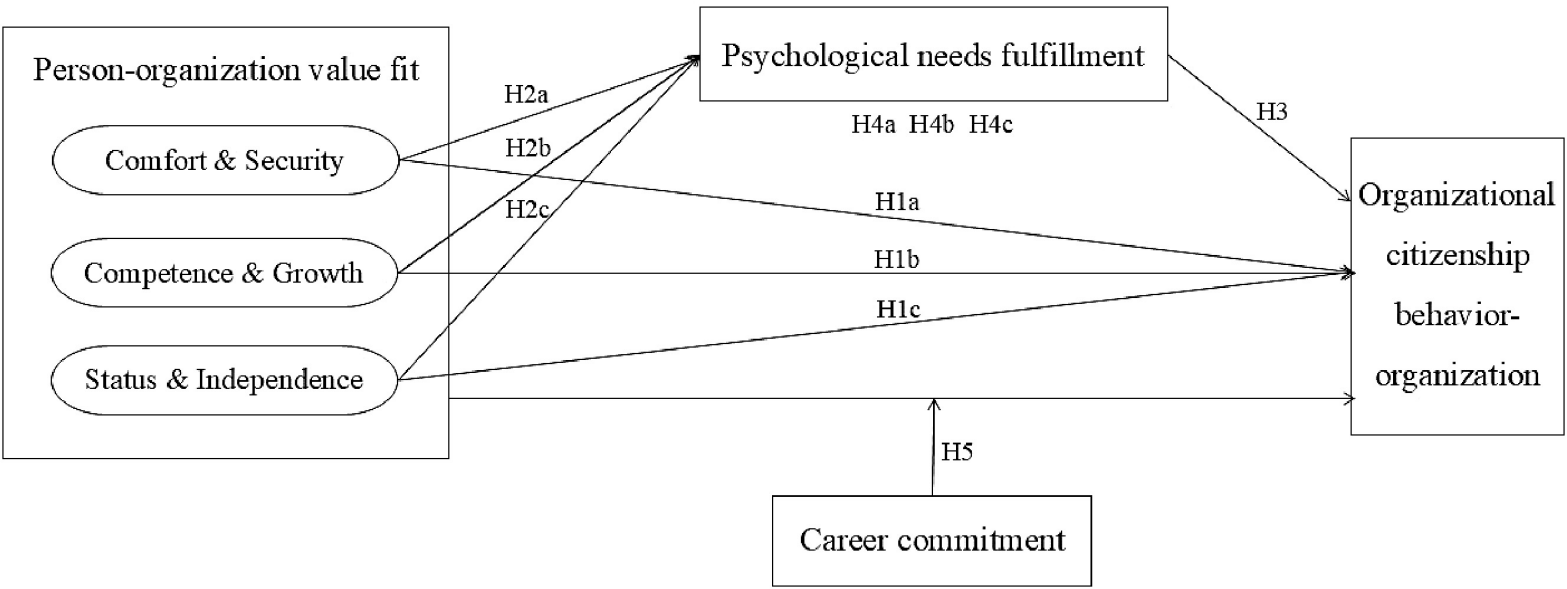
Research framework.

Taken together, the objective of this study is to establish a model that delves into the effects of three dimensions of POVF -comfort and security, competence and growth, status and independence- of the post-90s employees on their OCBO, which is directly beneficial to the organization (Ashfaq and Hamid, 2021), based on the view within SDT. Additionally, this study aims to investigate the underlying mechanisms and boundary conditions contributing to this relationship by examining the mediating effect of PNF and the moderating effect of CC. The research framework is illustrated in Figure 1.

This study contributes to the existing body of literature in several ways. Firstly, it investigates the impact of various dimensions of POVF. Comparing the different effects of POVF on employees’ psychological and behavioral outcomes enriches empirical evidence in the current literature available on the topic and provides new insight for future researchers. Secondly, previous research into the link between POVF and OCBO has been lacking in elucidating impact mechanisms from the perspective of psychological needs fulfillment. Therefore, investigating PNF as a potential mediator contributes significantly to deepening the comprehension of the psychological processes underlying the relationship, and enriching the theoretical boundary of SDT. Thirdly, this study examines the new moderate role of career commitment, extending the boundary condition regarding the relationship to be investigated. Moreover, the results of this study have significant implications for organizations that aim to achieve sustainable development and optimal utilization of human resources, inspiring the integration of value management with HRM and providing guidance for managerial strategies that promote employees’ extra-role beneficial behaviors.

## 2 Theoretical background and hypothesis development

### 2.1 Organizational citizenship behavior-organization

Bateman and Organ (1983) were the first to propose the concept of OCB. Building upon this foundation, Organ (1988) further refined the conceptualization of OCB as “individual behavior that is discretionary, not directly or explicitly recognized by the formal reward system, and that in the aggregate promotes the effective functioning of the organization” (p. 4). Among the numerous concepts and descriptions of OCB, three core characteristics can be summarized: (1) it is voluntary behavior of employees; (2) there is no formal reward system; and (3) it helps improve the effectiveness of the organization. These three core features of OCB have been widely recognized in academic literature. Williams and Anderson (1991) classified OCB as organizational citizenship behavior-individual (OCBI) and organizational citizenship behavior-organization (OCBO), defining OCBI as employees’ voluntary behaviors that are helpful to co-workers, and OCBO as behaviors beneficial to the organization.

### 2.2 Person-organization value fit

Chatman (1989) and Edwards and Cable (2009) possess analogous interpretations of the concept of POVF, who tend to define it as the extent of consistency or similarity between individual values and organizational values. Chung (2017) argued that the compatibility between an individual’s values and those of the organization can impact the individual’s work-related attitudes and behaviors. Previous studies have indicated that the younger generations of employees are characterized by an emphasis on work values such as self-achievement (Westerman and Yamamura, 2007), leisure, work-life balance, associated with status and compensation (Twenge et al., 2010). Based on these features, this study focused on three dimensions of work values, including comfort and security (CS), competence and growth (CG), and status and independence (SI), which were enhanced by Meyer et al. (1998) of the original work values inventory proposed by Manhardt (1972). The CS, CG, and SI dimensions of POVF refer to the alignment between employees’ values and those of their organizations regarding work conditions and job security, work variety and growth opportunities, work independence and possibilities for advancement, respectively.

### 2.3 Self-determination theory

SDT is a motivational framework suggesting that as employees’ motivation shifts from less self-determined (controlled) to highly self-determined (autonomous), they are expected to increasingly exhibit optimal functioning (Deci and Ryan, 2000), which refers to their demonstration of personal and interpersonal “growth and development in terms of employee wellbeing (e.g., positive emotions, vitality), attitudes (e.g., job satisfaction, organizational commitment), and behavior (e.g., performance, proactivity, and collaborative behaviors)” (Van den Broeck et al., 2019, p. 30). Based on meta-analytic evidence, motivation that is self-determined (autonomous motivation), is found to have a more positive correlation with attitudinal and behavioral outcomes (Van den Broeck et al., 2021). Autonomous motivation occurs when the basic psychological needs of autonomy, competence, and relatedness are satisfied (Deci and Ryan, 2000, Deci et al., 2001). According to SDT, the need for autonomy is the desire to feel psychologically free and possess a sense of control over one’s actions (i.e., a sense of self-organizing one’s behaviors). Competence needs reflect an individual’s natural inclination to explore and manipulate their environment and to pursue challenges that are optimal for their growth (i.e., a sense of mastery and effectiveness). The need for relatedness is fulfilled when individuals feel a sense of belonging to a group, establishing close relationships (i.e., a sense of connection and being significant to others).

### 2.4 Effect of person-organization value fit on organizational citizenship behavior-organization

As reviewed in 2.3, SDT suggests that employees perform better when motivated autonomously (Deci and Ryan, 2000). The compatibility between individual and organizational values engenders an environment conducive to fostering autonomous motivation among employees, motivating them to participate in positive behaviors (Zhang et al., 2010). When employees value comfort and security, or competence and growth, or status and independence of their work equally with their organizations, this alignment fosters the emergence of positive affective states, including trust and satisfaction, they are more likely to share emotional connections with the organization, leading to beneficial behaviors for the organization (Zhan et al., 2017). Chung (2017) argued that when individuals perceive a congruence between their values and those espoused by their organization, they are more inclined to participate in behaviors that are advantageous to the organization while less likely to engage in behaviors that may harm the organization. Based on the above arguments and empirical evidence, we propose the following hypotheses:

*H1*: The (a) comfort and security, (b) competence and growth, (c) status and independence dimension of person-organization value fit is positively related to OCBO.

### 2.5 Effect of person-organization value fit on psychological needs fulfillment

According to the basic psychological needs theory, a core sub-theory of SDT, there exist three basic psychological needs that are crucial for human progress–autonomy, relatedness, and competence (Deci and Ryan, 2000). Environmental elements, such as the degree of congruence between individual attributes and contextual factors, can either facilitate or impede the satisfaction of psychological needs (Deci et al., 2017). This indicates that when employees are employed within organizations characterized by congruent values, such organizations are more inclined to foster environments that facilitate the fulfillment of employees’ three basic psychological needs (Liu et al., 2023).

Firstly, the CS dimension of POVF is reflected in the congruence of values about work conditions and job security (Meyer et al., 1998). Organizations that prioritize work comfort and security provide regular work routines and ample leisure time off the job, which fulfill the needs for autonomy of employees who place a parallel emphasis on these values by enabling them to have more autonomy in managing their time. Van den Broeck et al. (2016) found that job insecurity negatively correlates with autonomy, competence, and relatedness needs, indicating that job security is significant for the three fundamental needs. Additionally, the congruence of values on job security and work comfort can foster employees’ sense of belonging. The satisfaction of the need for relatedness is contingent upon individuals experiencing a sense of connectedness, belongingness, and close relationships within a group (Deci and Ryan, 2000; Hu et al., 2019).

Secondly, the CG dimension of POVF refers to the value congruence on factors such as work variety, creativity, development, and growth (Meyer et al., 1998). Organizations value development and growth will equip employees with structured learning and programs to augment knowledge and skills. If employees also value growth, the enhancement of knowledge, skills, and abilities will contribute to an increased sense of self-efficacy and a heightened perception of competence among them (Gagné et al., 2022). Moreover, task variety has a positive promoting effect on fulfilling competency needs (Gagné et al., 2022). Van den Broeck et al. (2016) indicated that the relations between cognitive demands and competence and relatedness were found to be positive because cognitively demanding jobs may represent challenges for employees and be more likely to require teamwork, leading to an increase in competence and relatedness.

Thirdly, the SI dimension of POVF refers to the value alignment regarding work independence and opportunities for promotion (Meyer et al., 1998). Specifically, if both employees and the organization value these values, independent work, opportunities for promotion, and responsibility for significant work and risks can exert a positive effect on satisfying the needs for autonomy and competence. According to Messmann et al. (2022), fulfilling employees’ needs for autonomy can be achieved by granting them responsibilities and choices related to meaningful tasks and decisions. Additionally, position and status are significant determinants of respect from others, which play a positive role in fulfilling one’s need for relatedness. Therefore, based on the above arguments and empirical evidence, we propose the following hypotheses:

*H2*: The (a) comfort and security, (b) competence and growth, (c) status and independence dimension of person-organization value fit is positively related to psychological needs fulfillment.

### 2.6 Effect of psychological needs fulfillment on organizational citizenship behavior-organization

SDT suggests that optimal motivation in humans is contingent upon the satisfaction of the three fundamental psychological needs: autonomy, competence, and relatedness (Deci and Ryan, 2000). OCBs can be motivated by perceived needs satisfaction, as it provides the necessary intrinsic motivation for employees to showcase prosocial behaviors (Wörtler et al., 2020). Furthermore, when employees perceive a sense of autonomy, competence, and relatedness at work, it engenders the motivational energy essential for the activation of their inclination toward the internalization of external values and norms, which fosters the development of a self-determined motivation toward positive behaviors (Messmann et al., 2022; Rigby and Ryan, 2018). Based on multiple research evidence, Chen et al. (2020) posited that PNF of autonomy, competence, and relatedness stimulates intrinsic motivation and internalization of extrinsic motivation, which in turn affects individual behaviors, including positive behaviors, such as OCBO (Van den Broeck et al., 2016). Moreover, when employees’ needs for relatedness are satisfied, it enhances their sense of connection to others or groups. Research supports the proposition that individuals who perceive themselves as belonging to a group within their organization or the organization itself are more inclined to participate in OCBO (Chiniara and Bentein, 2016). Thus, we propose the following hypothesis:

*H3*: Psychological needs fulfillment is positively related to OCBO.

### 2.7 The mediation effect of psychological needs fulfillment

Greguras and Diefendorff (2009) suggested that fit influences employees’ attitudinal and behavioral outcomes through the fulfillment of needs. According to Zhang et al. (2010), the three basic psychological needs are a pivotal link between the external environment and individual motivation and behavior. When employees are employed within organizations characterized by congruent values, such organizations are more inclined to foster environments that facilitate the fulfillment of employees’ three basic psychological needs (Liu et al., 2023). The CS, CG, and SI dimensions of POVF, respectively, foster an environment conducive to meeting the fundamental psychological needs of employees as derived in 2.5. Further, such satisfaction of the three basic needs nurtures motivation for behaviors that are beneficial to the organization (Sedlářík et al., 2024). In light of the above arguments, we formulate the following mediation hypotheses:

*H4*: Psychological needs fulfillment will mediate the relationship between (a) comfort and security, (b) competence and growth, (c) status and independence dimension of person-organization value fit and OCBO.

### 2.8 The moderating effect of career commitment

According to Blau (1985) and Lee et al. (2000), the principal manifestation of career commitment is emotions that lie in the individual’s affection toward their current vocation and their aspiration to persist in it. Based on another sub-theory of SDT, the organismic integration theory, which introduced the concept of internalization of extrinsic motivation (Deci and Ryan, 1985). When an individual realizes the value of instrumental goals, there is an internalization process of extrinsic motivation, in which extrinsic motivation is progressively assimilated into autonomous motivation (Ryan and Deci, 2000). Autonomous motivation is significant for promoting individual optimal functioning, including positive attitudinal and behavioral outcomes (Van den Broeck et al., 2021). When employees exhibit a high level of commitment toward their careers, they are more inclined to discern the meaning of the work itself and recognize the inherent value of the work they undertake. This is crucial for stimulating autonomous motivation toward work, especially the identified regulation, which refers to the internalized process of extrinsic motivation through identifying the significance and meaningfulness of one’s actions (Howard et al., 2018). Grounded in the motivation theory within SDT, the alignment of individual and organizational values positively influences OCBO by cultivating an environment that fosters autonomous motivation among employees (Zhang et al., 2010). Therefore, CC could potentially have a significant moderating effect on the relationship between POVF and OCBO through the enhancement of autonomous motivation, thereby we propose the following hypothesis:

*H5*: Career commitment positively moderates the relationship between POVF and OCBO that the effect will be stronger when the level of career commitment is higher.

## 3 Materials and methods

### 3.1 Data collection

This research has been conducted in the form of a survey, with data collection being distributed among post-90s employees working in diverse industries and institutions in China. Given that this research was conducted in an undocumented market where secondary data is unavailable, thus, as the primary source, data questionnaires have been chosen, which is considered the most reliable source of information. Additionally, the post-90s generation in China has typical traits due to most of them growing up as only children during a time of rapid economic development; they tend to be self-centered and strong-minded (Liu et al., 2023), which determines their weaker collectivism. Therefore, selecting this specific group as the research object has great theoretical and practical significance.

We have employed a combination of field and online surveys, with field research conducted in Guizhou Province and online research distributed in different cities. A random sampling approach has been employed to select organizations to ensure a diverse range of industries and institutions, such as education, healthcare, finance, and hospitality, were included. Convenience sampling was utilized to ensure that the samples were representative of the post-90s employees. We have collected 713 questionnaires, of which 639 were deemed valid after filtering by deleting questionnaires with too short response times, questionnaires with blank spaces, and several that do not meet the age requirements (with an effective recovery of 89.6%). Table 1 presents all demographic characteristics for the valid sample in detail. Based on the overall descriptive statistical distribution of the sample population characteristics, it becomes evident that the proportionate distribution of the sample data within this research is reasonable and possesses a certain degree of data representativeness.

**TABLE 1:** Demographic characteristics of research sample (*N* = 639).

Demographic factor	Category	Frequency	(%)
Gender	Males	274	42.9%
	Females	365	57.1%
Age	18–27	294	46.0%
	28–34	345	54.0%
Education level	Bachelor	338	52.9%
	Master	142	22.2%
	Doctor	25	3.9%
	Other	134	21.0%
Work experience	Under 1 year	86	13.5%
	1–5 years	275	43.0%
	6–8 years	155	24.3%
	Over 8 yeas	123	19.2%
Position	Regular	454	71.0%
	Managerial	185	29.0%
Income	Under 3,000 RMB	38	5.9%
	3,001–6,000 RMB	303	47.4%
	6,001–10,000 RMB	191	29.9%
	Over 10,000 RMB	107	16.7%

### 3.2 Measures

The measuring instruments of this research were all adopted or adapted from mature scales with high reliability and validity. Given that the original scales were developed in English, the present study, conducted in China, necessitated the translation of all scale items. To enhance the quality of the translation, a preliminary survey was conducted on a small scale (30 questionnaires), following which improvements were made to the translation based on the feedback received. The final translated items enabled the semantic integrity of the original items and their comprehensibility for the respondents. All items were rated using a 5-point Likert scale, ranging from 1 (strongly disagree) to 5 (strongly agree).

#### 3.2.1 Person-organization value fit

We considered subjective measures as more proximate indicators of employee attitudes and behaviors in comparison to objective measures as how individuals perceive fit has been argued to be the primary factor influencing employee attitudes and behaviors (Cable and DeRue, 2002). Therefore, this research adopted a direct measurement to assess POVF. The scale was adapted from the work values by Meyer et al. (1998), comprising three dimensions: comfort and security with 5 items, the sample item is “My organization provides comfortable working conditions, which is aligned with what I value,” competence and growth with 7 items, the sample item is “My organization encourages continued development of knowledge and skills, which is aligned with what I value,” status and independence with 6 items, the sample item is “My organization permits advancement to high administrative responsibility, which is aligned with what I value.”

#### 3.2.2 Organizational citizenship behavior-organization

OCBO was measured on an 8-item scale adopted from Lee and Allen (2002), which was clearly beneficial to the organization and avoided possible overlap with workplace deviance behavior (Lee and Allen, 2002; Robinson and Bennett, 1995). The sample item is “I attend functions that are not required but that help the organizational image.”

#### 3.2.3 Psychological needs fulfillment

PNF was assessed using 14 items adapted from the Work-Related Basic Need Satisfaction scale by Van den Broeck et al. (2010), which has been recommended due to avoiding content validity issues (Van den Broeck et al., 2016). The scale consists of three dimensions: need for autonomy with 5 items, the sample item is “I feel like I can be myself at my job,” need for competence with 4 items, the sample item is “I have the feeling that I can even accomplish the most difficult tasks at work,” need for relatedness with 5 items, the sample item is “At work, I feel part of a group.”

#### 3.2.4 Career commitment

The scale of career commitment developed by Blau has been widely utilized by researchers. CC was assessed by adapting from a revised measure of Blau (1988) by Cohen (2007), consisting of 6 items. The sample item is “I like this vocation too well to give it up.”

### 3.3 Data analysis

Firstly, the reliability and validity of the measurement instruments were examined based on confirmatory factor analysis (CFA), Cronbach’s alpha, composite reliability (CR), average variance extracted (AVE), utilizing SPSS 27 and AMOS 28 (Benitez et al., 2020; Fornell and Larcker, 1981; Hair et al., 2017). Secondly, structural equation modeling (SEM) was used to estimate the path coefficients among constructs using AMOS 28. Thirdly, we applied the Bootstrap approach with a 95% confidence interval to test the mediating hypotheses (Bollen and Stine, 1990). Lastly, the moderating effect was examined using PROCESS 4.2 as a complementary analytical approach. Although moderation can also be tested within a structural equation modeling framework through latent interaction techniques, such approaches typically involve more complex estimation procedures and may present challenges in terms of model convergence and interpretability in covariance-based SEM software. Given that the primary purpose of the moderation analysis in this study was to estimate and interpret interaction effects and conditional relationships, a regression-based approach was adopted.

## 4 Results

### 4.1 Common method bias test

In this study, we conducted two tests for common method bias. Firstly, based on Harman’s single-factor methods, our analysis revealed six factors with eigenvalues greater than 1, collectively explaining 62% of the total variance. Notably, the first factor explained 17% of the cumulative variance, falling below the 40% threshold and not surpassing half of the total variance explained. This indicated that this research was not significantly affected by common method bias.

In addition, controlling for the effects of an unmeasured latent methods factor (ULMC) was employed (Podsakoff et al., 2003), a widely recognized robust technique for detecting common method variance. The absence of a significant change in model fit between the two-factor model and the original model indicates a lack of substantial common method bias (Podsakoff et al., 2012). In the present study, comparison of model fit indices revealed minor differences (△ RMSEA = 0.013, △ CFI = 0.005, △ GFI = 0.008, △ NFI = 0.007, △ TLI = 0.005), indicating that the model did not exhibit significant improvement after the inclusion of the common method factor. Therefore, further verification of common method bias does not pose a serious problem in this study.

### 4.2 Confirmatory factor analysis

The KMO and Bartlett’s test of sphericity result was 0.964 (*p* < 0.000), demonstrating the validity of the sample content is good for engaging in further factor analysis. SPSS 27 and AMOS 28 were used to conduct confirmatory factor analysis (CFA) to assess the internal reliability, convergent validity and discriminant validity of the model. The internal reliability was examined by Cronbach’s alpha and composite reliability (CR). As recommended, a Cronbach’s alpha of 0.7 and above indicates adequate reliability, and a CR value between 0.7 and 0.95 is considered reliable (Hair et al., 2017). As reported in Table 2, the Cronbach’s alpha coefficient of all structures ranged from 0.848 to 0.936, and the CR values of all constructs ranged from 0.849 to 0.936, confirming adequate reliability.

**TABLE 2:** Confirmatory factor analysis and reliability and validity indicators.

Constructs	Cronbach’s α (> 0.7)	CR ( > 0.7)	Factor loading (> 0.5)	AVE ( > 0.5)
CS	0.848	0.849	0.688–0.755	0.530
CG	0.910	0.910	0.749–0.788	0.592
SI	0.880	0.881	0.717–0.782	0.551
OCBO	0.917	0.917	0.739–0.794	0.581
PNF	0.936	0.936	0.676–0.769	0.512
CC	0.901	0.901	0.759–0.816	0.603

CR, composite reliability; AVE, average variance extracted.

The convergent validity was examined by standardized factor loading and average variance extracted (AVE). The AVE score of each construct over 0.5 shows good convergent validity (Fornell and Larcker, 1981). As presented in Table 2, all factor loadings were higher than 0.6, and the AVE score of all constructs satisfied the lowest level of 0.5, indicating good convergent validity. As per Fornell and Larcker (1981), the AVE of all constructs should exceed the squared value of the correlation coefficient between two constructs to demonstrate discriminant validity. As shown in Table 3, the square root values of the AVE of each construct surpassed the correlation coefficient between the two constructs. This outcome substantiated the adequate discriminant validity of the constructs under examination.

**TABLE 3:** Discriminant validity indicators.

Constructs	CS	CG	SI	PNF	OCBO	CC
CS	1	1	1	1	1	1
CG	0.415					
SI	0.378	0.488				
PNF	0.362	0.406	0.381			
OCBO	0.439	0.509	0.496	0.404		
CC	0.300	0.337	0.355	0.348	0.676	
Square root of AVE	0.728	0.769	0.742	0.762	0.716	0.777

### 4.3 Path coefficients test

By conducting SEM in AMOS 28, the overall fit indices (χ^2^/df = 1.175 < 3; GFI = 0.937 > 0.9; AGFI = 0.929 > 0.9; NFI = 0.941 > 0.9; TLI = 0.990 > 0.9; CFI = 0.991 > 0.9; RMSEA = 0.017 < 0.08) all surpassed the criterion (Hu and Bentler, 1999), as reported in Table 4, indicating a good fit for the structural model.

**TABLE 4:** Fit indices, criterion and values of the structural model.

Model fit index	Model fit criterion	Model fit values	Good fit (Y/N)
χ^2^/df	< 3	1.175	Y
GFI	> 0.9	0.937	Y
AGFI	> 0.9	0.929	Y
NFI	> 0.9	0.941	Y
TLI	> 0.9	0.990	Y
CFI	> 0.9	0.991	Y
RMSEA	< 0.08	0.017	Y

χ^2^, Chi-square; df, degree of freedom; GFI, goodness-of-fit index; AGFI, adjusted goodness-of-fit index; NFI, normed fit index; TLI, Tucher-Lewis index; CFI, comparative fit index; RMSEA, root mean square error of approximation.

The hypothesis testing and path effect analysis were executed utilizing AMOS 28, with the outcomes delineated in Table 5. The three dimensions of POVF -comfort and security, competence and growth, status and independence- have a positive influence on OCBO, with significant *p-*values (β = 0.211, *p* < 0.001, β = 0.256, *p* < 0.001, β = 0.268, *p* < 0.001). Therefore, H1(a), H1(b), and H1(c) are accepted. Additionally, these three dimensions, the CS, CG, and SI dimensions of POVF, are discovered to exert a significant positive effect on PNF, respectively (β = 0.206, *p* < 0.001, β = 0.237, *p* < 0.001, β = 0.201, *p* < 0.001). Thus, H2(a), H2(b), and H2(c) are supported. Lastly, the fulfillment of psychological needs has been proven to have a significant and positive correlation with OCBO (β = 0.123, *p* < 0.01), thereby lending support to Hypothesis 3.

**TABLE 5:** Path results of SEM.

Hypotheses	Path	Std.estimate (β)	S.E.	C.R.	*p*-value	Results
H1a	CS → OCBO	0.211	0.059	4.72	***	Supported
H1b	CG→ OCBO	0.256	0.055	5.43	***	Supported
H1c	SI → OCBO	0.268	0.059	5.72	***	Supported
H2a	CS → PNF	0.206	0.048	4.25	***	Supported
H2b	CG → PNF	0.237	0.044	4.65	***	Supported
H2c	SI → PNF	0.201	0.047	4.00	***	Supported
H3	PNF → OCBO	0.123	0.054	3.04	**	Supported

***Indicates *p* < 0.001, **Indicates *p* < 0.01, *Indicates *p* < 0.05.

### 4.4 Mediating effect test

We employed the Bootstrap approach (number of samples = 5,000) with a 95% confidence interval (CI) to examine the mediating effect of PNF. As reported in Table 6, the total effects of the CS, CG, and SI dimensions of POVF on OCBO were 0.237, 0.285, and 0.293, respectively. The indirect effects of the CS, CG, and SI dimensions of POVF on OCBO via PNF were 0.025 [(95 % CI = (0.007, 0.054)], 0.029 [95 % CI = (0.007, 0.065)], and 0.025 [95% CI = (0.007, 0.055)] respectively. All the mediation test results were significant, indicating that the impacts of the CS, CG, and SI dimensions of POVF on OCBO are partially mediated by PNF. Therefore, H4(a), H4(b), and H4(c) are accepted.

**TABLE 6:** Mediated path coefficient.

Hypotheses	Path	Total effect	Indirect effect	Bootstrap 95% CI		Results
				Lower	Upper	
H4a	CS → PNF→ OCBO	0.237	0.025	0.007	0.054	Supported
H4b	CG → PNF→ OCBO	0.285	0.029	0.007	0.065	Supported
H4c	SI → PNF→ OCBO	0.293	0.025	0.007	0.055	Supported

CI, confidence interval.

### 4.5 Moderating effect test

**FIGURE 2:**
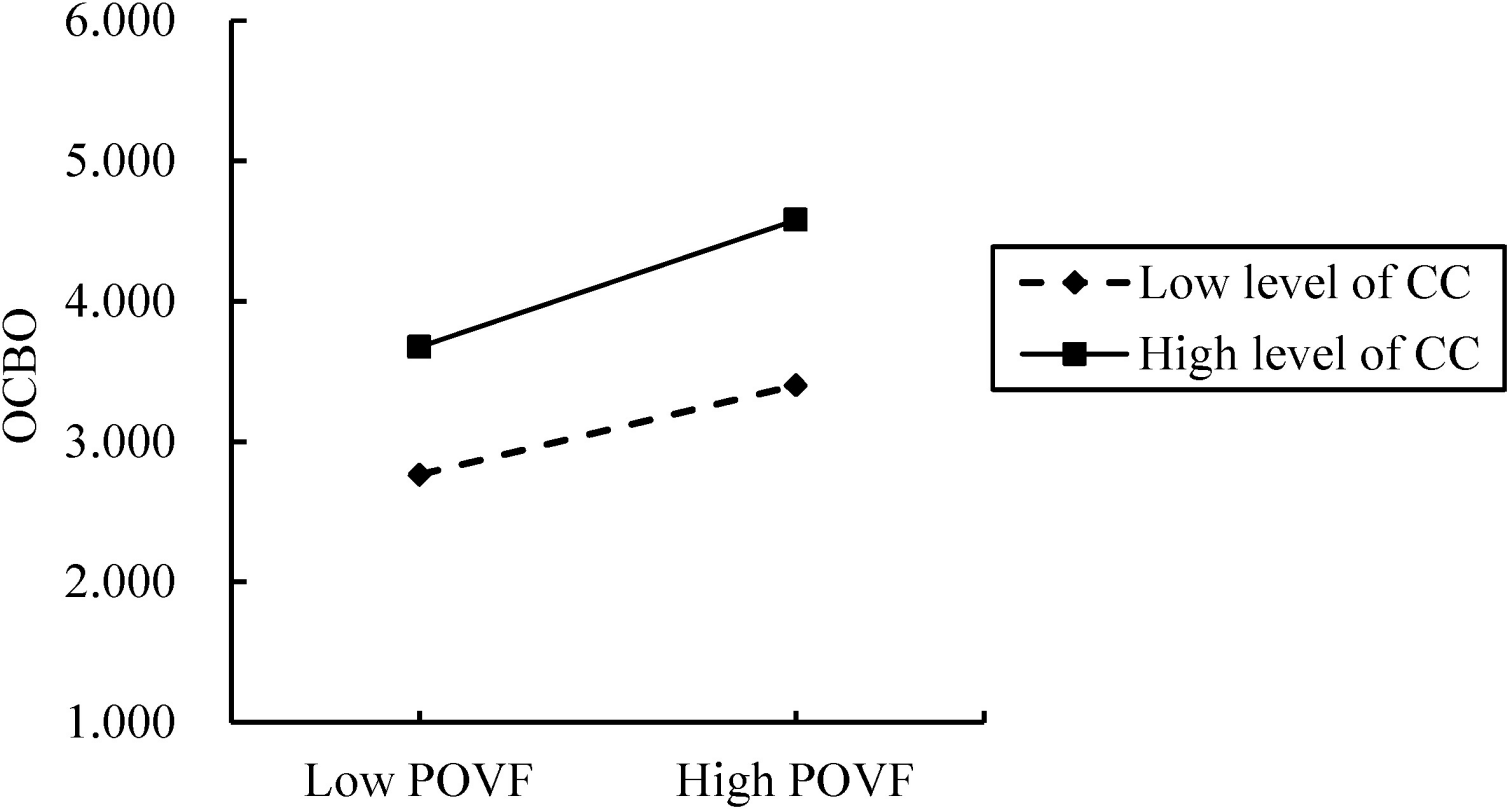
The moderation effect of career commitment on the relationship between person-organization value fit and organizational citizenship behavior-organization.

In this study, we investigated the moderating role of CC on the relationship between POVF and OCBO, employing PROCESS 4.2 for analysis. The Bootstrap (number of samples = 5,000) results revealed that the interaction coefficient was 0.012 (*p* < 0.01, S.E. = 0.038, *t* = 2.65), with a 95% CI of (0.026, 0.177). This finding indicated a statistically significant moderating effect of CC. Specifically, it elucidated that CC enhances the influence of POVF on OCBO, that as the level of CC increases, the impact of POVF on OCBO becomes more pronounced, as shown in Figure 2. Therefore, Hypothesis 5 is accepted.

## 5 Discussion

### 5.1 Theoretical implications

This study makes a valuable contribution to the existing body of literature in several ways. Firstly, our findings indicated that POVF exerts a positive influence on OCBO. When an individual’s values align with those of the organization they work for, it creates a positive impression and higher evaluations of the organization, resulting in behaviors advantageous to the organization (Zhan et al., 2017). The result concurs with the perspectives and results presented by Ashfaq and Hamid (2021) and Zoghbi-Manrique-de-Lara et al. (2023). In contrast, our study examined the impact of various dimensions of POVF on OCBO, setting itself apart from previous research. Based on the work values that are emphasized by Millennial and Gen Z employees, we compared the impact of three dimensions of value fit on their out-of-role behaviors that contribute to the organization.

The findings indicate that the status and independence dimension of POVF exerts the most significant influence on OCBO, followed by the competence and growth dimension, and lastly the comfort and security dimension. Drawing upon Maslow’s Hierarchy of Needs, the value fit associated with the comfort and security dimension satisfies lower-order needs, whereas the fit with competence and growth, as well as status and independence, fulfills higher-order needs. The extent to which higher-level needs are met is positively correlated with increased motivation, thereby further facilitating OCB (Hidayah and Hendarsjah, 2021). It is noteworthy that, although variations exist in the effects of different dimensions on OCBO in this study, these differences are very minor. This indicates a stable influence of POVF on OCBO that is not significantly impacted by dimensions, indirectly substantiating the robustness of this relationship. This stable relationship can be explained as an individual’s positive perception of his/her surrounding environment is positively correlated with an increased propensity for exhibiting positive behavior (Udin, 2020). Value alignment, regardless of the dimension, is beneficial for creating such a positive perception.

Secondly, the results of this study indicated that PNF serves as a partial mediator in the nexus between the comfort and security, competence and growth, and status and independence dimensions of POVF and OCBO. These findings have validated previous arguments that the fulfillment of the basic psychological needs as a “nutriment” promotes positive behaviors and attitudes among individuals (Deci and Ryan, 2000; Ryan and Deci, 2000), just as “plants need water, sunshine, and minerals to thrive” (Van den Broeck et al., 2016, p. 1198). The CS, CG, and SI dimensions of POVF provide support for satisfying the three basic psychological needs, fostering autonomous motivation, which in turn affects individual attitudes and behaviors (Van den Broeck et al., 2016). Prior research investigating the influence of POVF on OCBO has predominantly focused on the mediating roles of employees’ attitudinal factors, such as work engagement (Ashfaq and Hamid, 2021) and employees’ compassion at work (Zoghbi-Manrique-de-Lara et al., 2023). This study is grounded in autonomous motivation theory within SDT, elucidating the mediating role of basic psychological needs in linking person-organization value fit to employees’ positive behavior. Thus, this study contributes to the literature by focusing on autonomous motivation as the primary driver, explaining how fulfilling psychological needs acts as a facilitative mechanism from a new perspective.

Thirdly, this study discovered that CC plays a moderating role in influencing the relationship between POVF and OCBO that the effect of POVF on OCBO is stronger when the level of employees’ commitment to their careers is higher. This finding has provided empirical support for previous arguments that when employees exhibit high levels of career commitment, they are more likely to be motivated to make valuable contributions to organizational success (Ahmed, 2019). POVF stimulates employees’ autonomous motivation (Zhang et al., 2010), thereby promoting their OCBO (Van den Broeck et al., 2021). While a prominent level of CC strengthens this impact by enhancing employees’ autonomous motivation. Our study has extended the boundary condition regarding this relationship between POVF and OCBO and has responded to the call for further research investigation of the moderators pertaining to this relationship (Ashfaq and Hamid, 2021).

Lastly, this study employed the self-determination theory as its theoretical underpinning to elucidate the correlation between POVF and OCBO, the mediating effect of PNF, and the moderating effect of CC. Specifically, we engaged with two sub-theories of SDT, namely basic psychological needs theory and organismic integration theory (Deci and Ryan, 2000; Deci et al., 2001; Ryan and Deci, 2000). We explained the mediating effect of PNF by drawing upon the auspices of basic psychological theory. Furthermore, organismic integration theory was applied as the theoretical underpinning to elucidate the moderating effect of CC. Therefore, this study has enriched the application of SDT and its sub-theories by employing them within a distinctive content of the impact mechanism of POVF on OCBO and a distinctive context of the Chinese post-90s employees. By doing so, it broadens the scope of SDT and makes a significant contribution to the advancement of the theory.

### 5.2 Practical implications

The results of this study have significant implications for organizations. Firstly, this study suggests that POVF is positively related to employees’ OCBO, which gives corporations managerial implications that if they want to promote employee extra-role behaviors that are beneficial to the organization, an effective way is to ensure organizational values align with those of employees. As the congruence of values is a dynamic process that may evolve over time (Swider et al., 2015; Vleugels et al., 2019), managing to promote the alignment of values is more crucial for organizations. Especially when facing the younger generations of employees, who have greater information reception ability and stronger value judgment, value management could be a new and critical strategic tool to attain sustained competitiveness for organizations (Zhan et al., 2017). Given the diversity in employees’ work value orientations, organizations should acknowledge and respect these differences, developing incentive policies that align with their preferences to motivate their initiative for work. Specifically, to assign positions with regular working routines in time and place to employees who place a high emphasis on the CS dimension values; to offer a diverse array of tasks along with opportunities for the development of knowledge and skills to employees who prioritize the CG dimension values; responsibilities for risk-taking, coupled with avenues for promotional advancement should be extended to employees who value the SI dimension values (Ren et al., 2021).

Secondly, the findings of this study reveal that PNF is a stimulus by which POVF affects OCBO. Thus, companies can significantly enhance employees’ OCBO by paying extra attention to this mediator. It is crucial for organizations to cultivate a positive culture that satisfies employees’ psychological needs, such as management styles that support autonomy, reward structures or performance systems that deliver feedback regarding one’s competency, and organizational activities that meet relatedness needs (Greguras and Diefendorff, 2009). In general, organizations can address employees’ basic psychological needs by endowing them with work-related autonomy, providing them with opportunities for growth and development, nurturing a supportive network, and promoting a cohesive organizational culture.

Thirdly, the moderating effect also provides direction for management. The younger generation of employees tends to develop more commitment to their own careers than to their organizations (De Hauw and De Vos, 2010; Zhu et al., 2021). Therefore, to augment the degree of dedication of the younger generation employees to an organization, it is significant for organizations to improve their sense of career commitment. According to Ahmed (2019), employees are more likely to remain loyal and committed to their careers if their organization can fulfill their career needs. Based on a meta-analytic review of the antecedents of career commitment, Zhu et al. (2021) have identified numerous factors that impact career commitment. Among them, organizational factors include promotion opportunities and career growth. Specifically, career commitment can be achieved by establishing a robust career development platform, serving employees by augmenting their career adaptability, bolstering their career competencies and proficiencies, and creating more opportunities for career growth.

### 5.3 Limitations and avenues for future research

The current study has certain limitations that offer directions for future research. The research data were obtained from China, so the results may have regional limitations due to cultural and background differences. Moreover, since the study only surveyed employees born since the year 1990, the research results may have limited applicability to other generations. Future research can investigate the interrelationship among POVF, PNF, CC, and OCB across diverse generational cohorts and geographical locations. Additionally, the data in this study are subject to certain limitations. The use of cross-sectional and self-reported data may introduce common method bias. Although two types of common method bias tests were implemented, the results indicated that this research was not significantly affected by common method bias, as presented in 4.1. Future research can employ longitudinal designs and multiple raters to assess variables such as OCB.

## 6 Conclusion

The present study discussed the influence of various dimensions of person-organization value fit on employees’ organizational citizenship behavior toward organizations, considering the mediation role of psychological needs fulfillment and exploring the moderating effect of career commitment. The findings suggest that various dimensions of value congruence exert similar positive effects on OCBO, thereby substantiating the stable nexus between these constructs. Our research enriches the existing literature by providing significant empirical evidence for the underlying mechanisms and boundary conditions for this relationship from a novel perspective. Furthermore, it provides crucial practical implications for organizations aiming to optimize human resource utilization effectively.

## Data Availability

The raw data supporting the conclusions of this article will be made available by the authors, without undue reservation.
